# Genetic and physiological variation in two strains of Japanese quail

**DOI:** 10.1186/s43141-020-00100-3

**Published:** 2021-01-20

**Authors:** Nashat Saeid Ibrahim, Mohammed Ahmed El-Sayed, Heba Abdelwahab Mahmoud Assi, Ahmed Enab, Abdel-Moneim Eid Abdel-Moneim

**Affiliations:** 1 Biological Application Department, Nuclear Research Center, Egyptian Atomic Energy Authority, P.O. Box 13759, Cairo, Egypt; 2 National Gene Bank, Animal Genetic Resources Department, Agricultural Research Center, Giza, Egypt; 3 Animal Production Research Institute, Agricultural Research Center, Giza, Egypt; 4 Department of Poultry and Fish Production, Faculty of Agriculture, Menoufia University, Shibin El Kom, Egypt

**Keywords:** Japanese quail strain, Genetic and physiological variation, Microsatellite markers

## Abstract

**Background:**

Detecting the genetic and physiological variations in two Japanese quail strains could be used to suggest a new avian model for future breeding studies. Consequently, two estimations were performed on two Japanese quail strains: gray quail strain (GJQS) and white jumbo quail strain (WJQS). The first estimation was conducted on carcass characteristics, breast muscles, breast concentration of collagen type I, and body measurements. In contrast, blood samples were collected for the second estimation for genomic DNA extraction and genetic analysis.

**Results:**

A total of 62 alleles out of 97 specific alleles (63.92%) were detected overall loci (14 microsatellite loci) for the two strains. A total of 27 specific alleles of WJQS were observed, and 35 were obtained for GJQS. The percentage of similarity was 48.09% ranged from 4.35 with UBC001 to 100% with GUJ0051. WJQS had greater body weights and a higher value of pectoral muscle and supracoracoideus muscle than GJQS. The breast muscles of GJQS exhibited a higher concentration of type I collagen than the WJQS. Furthermore, males showed higher concentrations of collagen type I than females. WJQS showed a higher body length, chest girth, chest length, thigh length, thigh girth, drumstick length, and drumstick girth (cm) than GJQS. WJQS showed more significant differences in carcass traits compared with GJQS.

**Conclusion:**

The physiological differences between WJQS and GJQS were ascertained with microsatellite markers, which indicated high polymorphism between these strains. These observations provided a scientific basis for evaluating and utilizing the genetic resources of WJQS and GJQS in a future genetic improvement program.

## Background

Japanese quail (*Coturnix coturnix japonica*) is currently the smallest poultry species reared primarily for meat and egg production [1]. It has unique characteristics, including rapid growth, quick life cycle, disease resistance, early sexual maturity, high rate of lay, and lower feed consumption [2, 3]. These characteristics significantly differ between the Japanese quail strains. Therefore, quails were divided into different strains according to breeding, either meat production quails, egg production quails, or dual-purpose quails. Besides, Mohammed et al. [4] reported various plumage color mutations in Japanese quails and white and gray plumage colors that may be considered different quail strains. Thus, it is essential to assess the existence of genetic and substantial physiological variations within these strains to establish effective breeding programs to improve the most important economic traits. Many studies have reported some estimates of genetic parameters for various traits of Japanese quail’s body and performance [5, 6]. They concluded that the continuous increase could improve the growth performance and egg production of the Japanese quail in their genetic potential and favorable management conditions. Hence, the characterization of indigenous bird populations’ physiological parameters and genetic diversity is a prerequisite tool for providing needed information for the conservation of useful genotypes to improve efficiency and significant productivity of birds [7, 8]. The mentioned characterization can be achieved using the microsatellite marker technique to estimate the variability and genetic relationships between and within the bird’s populations [9]. Habimana et al. [10] also evaluated the degree of genetic diversity and phylogenetic relationships between IC populations in Rwanda by using simple sequence repeats markers. Therefore, the purpose of this study was to characterize and detect the genetic and physiological variations in two strains of Japanese quails (gray quail strain (GJQS) and white jumbo quail strain (WJQS)) and to determine the molecular description for these strains by physiological measurements and molecular genetics, and lastly to suggest a new avian model for future breeding studies.

## Methods: birds’ husbandry and ethics

Two strains of Japanese quails (GJQS *n* = 60 and WJQS *n* = 62), at 5 weeks old, were maintained at the quail experimental farm of the Biological Application Department, Nuclear Research Center, Egypt. Birds were reared in battery cages of 100 × 60 × 50 cm (length × width × height) in size, categorized by each strain, and fed a diet matching with the National Research Council [11]. All procedures used in this investigation were approved by the scientific and ethics committee of the Biol. Appli. dep., (protocol number 187; date of approval: 28 August 2019), according to the policies and guidelines of the institutional poultry care and use committee.

### Collection of data

Two estimations were performed on GJQS and WJQS as follows:

#### Physiological estimations

Bird weights and biometric body measurements (cm) were collected individually for each strain using a flexible measuring tape. The biometric body measurements (cm) include body length, chest length, chest girth, thigh length, thigh girth, drumstick length, and drumstick girth. Birds were slaughtered, and the empty carcass, liver, heart, intestine, gizzard, proventriculus, and spleen were weighed, recorded, and expressed as a percentage of live body weight. Dressing percentage and carcass yield were estimated as described by Abd El-Moneim et al. [12] and Abdel-Moneim et al. [13]. The breast muscle was exposed, and both right and left supracoracoideus (SC) and pectoralis major muscle (PC) were excised, weighed, and expressed in absolute weight and relative weight. Samples of the breast muscle tissue 0.1 g were taken out and rinsed with 1x PBS, freeze-thaw cycles to break the cell membranes, and centrifuged for 5 min at 5000×*g*, 2–8 °C. The supernatant was removed, and the quantitative determination of collagen type I concentrations were determined immediately using the ELISA kit (catalog number csb-e0804r) produced by CUSABIO TECHNOLOGY LLC (http://www.cusabio.com), Houston, TX 77054, USA.

#### Genetic analysis

Blood samples were collected from GJQS (*n* = 20) and WJQS (*n* = 22), for genomic DNA extraction according to methods described by Sambrook et al. [14] as follows: a half milliliter of the blood sample was withdrawn from the jugular vein on EDTA tube as anti-coagulant (0.2 ml of 0.5 M EDTA). DNA was freshly extracted from the whole collected EDTA-blood. Two and a half milliliter of lysis buffer TSTM (20 mM Tris-HCl pH 7.6, 640 mM sucrose, 2% Triton X-100, 10 mM MgCl2) was added to the aliquot. The mixture was centrifuged, and the pellet was suspended in 150 μl proteinase K, 1.5 ml nuclei lysis buffer, and 110 μl SDS 20%. After overnight incubation at 37 °C, the proteins were removed by NaCl 6 M, and the DNA was precipitated by ice-cold absolute ethanol.

##### Microsatellite genotyping: source of primers

Fourteen primer pairs of microsatellite markers, as shown in Table 1 were designed according to the literature of Kayang et al. [15], Charati et al. [16], Moradian et al. [17], and Roushdy and El-Sayed [9]. Applying these locations specifically in the present study will explain the results of physiological estimations such as body weight, morphometric body measurements, carcass traits, breast muscle weight, and soluble collagen.

**Table 1:** Microsatellite loci used, annealing temperatures, primers sequence, gene bank accession numbers, and reported type and size range with jumbo and grey Japanese quail strains

Locus name	AT. (°C)	Primers sequence	Chromosome no.	Gene Bank accession no.	Repeat type	Band Size (bp)
GUJ0013	55	ACCAAACCCGAGATCCGACA AGCGTTCGCGTTCCTCTTTC	GGA1	AB035823	(CA)10	80–100
GUJ0021	62	GAGCATTTCTAGTCTGTCTC GATCAATACACAGGCTAAGG	CJA06	AB035831	(CA)11	155–188
GUJ0028	54.6	TGAACAAAGCAGAAAGGAGC CCTTACCTACATGAAACGTC	QL08	AB035838	(CA)9	104–167
GUJ0048	55	AACGCATACAACTGACTGGG GGATAGCATTTCAGTCACGG	CJA01	AB035858	(CA)14	52–94
GUJ0051	55	CCTTAACCACTCCTACTGAC TTTTGTAAGTGGCCCCGTAC	CJA01	AB063119	(CA)10	45–65
GUJ0052	55	AAACTACCGATGTAAGTAAG ATGAGATATATAAGGAACCC	CJA01	AB063120	(CA)12	55–151
GUJ0053	64	GCTGGAGTTTTACATGCACG TGGATTATGATGCTGACATAAG	Unknown	AB063121	(CA)19	177–215
GUJ0054	55	GTGTTCTCTCACTCCCCAAT ATGTGAGCAATTGGGACTG	CJA06	AB063122	(CA)7	54–103
GUJ0057	62	GGAATGGAAAATATGAGAGC CAGGTGTTAAAGTCCAATGT	CJA03	AB063125	(CA)12	130–250
GUJ0087	55	CATGCCGGCTGCTATGACAG AAGTGCAGGGAGCGAGGAAG	CJA06	AB063155	(CT)12AA(CA)11	154–198
GUJ0099	55	CTCTTATCCATCCTTCCTTC TTTTAAGTTTCCCCAGGCAG	CJA03	AB063167	(CA)16GA(CA)5(TA)7	35–77
UBC001	48	TCTCTAAAATCCAGCCCTAA AGCTCCTTGTACCCTATTGC	1	AF121113	(CAG)3 N9(CA)3TA(CA)5	475–610
UBC002	50	CAGCCAATAGGGATAAAAGC CTGTAGATGCCAAGGAGTGC	6	AF121114	(AT)3 T(CT)11A(AC)7	190–253
UBC005	57	GGAACATGTAGACAAAAGC AGTAGTCCATTTCCACAGCCA	3	AF121117	(AC)9	100–181

##### Polymerase chain reaction

The PCR was performed using 50–100 ng genomic DNA in a 25 μl reaction volume containing 10 μl Master Mix (Emerald AMP GT PCR Master Mix, Takara Bio. Inc. composed of 10 pmol of each primer, DNA polymerase, optimized reaction buffer, dNTPs and a density reagent). The premix also contained a vivid green dye, which is separated into blue and yellow dye fronts. The PCR reactions were carried out under the following conditions: an initial denaturation step (for 4 min at 95 °C), followed by 35 cycles of denaturation (for 1 min at 95 °C), annealing (at 48–64 °C for 1 min) at optimized primer annealing temperature (Table 1), and then extension (for 1 min at 72 °C) and final extension (for 10 min at 72 °C). Amplified fragments were analyzed on 10% polyacrylamide gel and stained with Ethidium bromide. The gels were photographed, and images were analyzed using the Gel Documentation System (Alpha imager TM 2200, Cell Biosciences).

### Statistical analysis

The physiological results were analyzed with the general linear model and variance procedure analysis between quail strains using the statistical software [18]. Tukey’s procedure for multiple comparison tests was used to identify significant differences of values at a significance level of 5%. All scored microsatellite data were firstly corrected to estimate each allele size according to its number of repeats for each marker. All possible extracted species’ figures were carried out employing an Arlequin 3.5 software package after data conversion using the CONVERT program. The POPGENE software package [19] was used to calculate allele frequencies, observed (HO) and expected (HE) heterozygosities, and ENA for WJQS and GJQS.

## Results

### Physiological estimation

#### Body measurements

Differences in body measurements between two Japanese quail strains are presented in Table 2. The WJQS showed a higher value in body length, chest girth, chest length, thigh length, thigh girth, drumstick length, and drumstick girth (cm) than GJQS. No significant differences were observed inside the sex strain in the mentioned body measurements.

**Table 2:** Differences in body measurements between Jumbo and Grey Japanese quail strains

Indices		Body length (cm)	Chest length (cm)	Chest girth (cm)	Thigh length (cm)	Thigh girth (cm)	Drumstick length (cm)	Drumstick girth (cm)
Quail strain	Gender							
Jumbo	Male	13.100	9.250	17.50	4.250	7.500	6.750	6.650
Jumbo	Female	13.000	9.175	17.75	4.625	7.650	6.750	6.875
Gray	Male	12.350	8.700	16.75	3.850	7.000	6.100	6.250
Gray	Female	12.250	9.150	16.50	4.250	6.350	5.500	6.350
SEM		0.138	0.178	0.214	0.130	0.203	0.195	0.108
Quail strain								
Jumbo		13.050^a^	9.213	17.625^a^	4.438	7.575^a^	6.750^a^	6.762^a^
Gray		12.300^b^	8.925	16.625^b^	4.050	6.675^b^	5.800^b^	6.300^b^
SEM		0.127	0.320	0.239	0.169	0.225	0.193	0.123
Gender								
Male		12.725	8.975	17.125	4.050	7.250	6.425	6.450
Female		12.625	9.163	17.125	4.438	7.000	6.125	6.612
SEM		0.110	0.277	0.207	0.146	0.195	0.167	0.106
Source of variation, *p* value								
Quail strain		0.004	0.522	0.020	0.133	0.023	0.010	0.029
Gender		0.575	0.673	1.000	0.133	0.433	0.285	0.356
Quail strain × gender		0.970	0.558	0.460	0.957	0.228	0.285	0.714

Means in the same column within each classification bearing different letters are significantly different

*SEM* Standard error of means

### Body weight and carcass characteristics

Variations between two Japanese quail strains in marketing body weight and carcass characteristics are recorded in Table 3. The WJQS had a larger body (312.7.0 vs. 279.3 g, *P* ˂ 0.001) weights compared with GJQS. The relative weight of carcass yield, dressing, liver, heart, proventriculus, and spleen except intestine and gizzard were significantly higher in WJQS than GJQS. Moreover, sex differences were observed inside strain itself, whereas male quail showed significant values in dressing, heart, and carcass yield percentages, while female quail showed significant values in marketing body weight, liver, intestine, proventriculus, and spleen percentages.

**Table 3:** Differences between jumbo and gray Japanese quail strains in marketing body weight and carcass characteristics

Indices		Body weight (g)	Dressing (%)	Liver (%)	Heart (%)	Intestine (%)	Gizzard (%)	Proventriculus (%)	Spleen (%)	Carcass yield (%)
Quail strain	Gender									
Jumbo	Male	296.0	75.96	1.673^c^	0.913	3.472^c^	2.026^a^	0.410	0.079	80.57
Jumbo	Female	329.3	68.93	2.275^a^	0.815	6.318^a^	1.795^b^	0.465	0.124	73.82
Gray	Male	254.7	71.21	1.187^d^	0.762	3.995^b^	1.934^ab^	0.314	0.056	75.09
Gray	Female	304.0	59.06	2.034^b^	0.644	6.061^a^	1.985^a^	0.335	0.084	63.72
SEM		8.320	1.936	0.125	0.030	0.377	0.033	0.019	0.008	1.919
Quail strain										
Jumbo		312.7^a^	72.44^a^	1.974^a^	0.864^a^	4.895	1.910	0.437^a^	0.101^a^	77.19^a^
Gray		279.3^b^	65.13^b^	1.611^b^	0.703^b^	5.028	1.959	0.324^b^	0.070^b^	69.41^b^
SEM		3.186	0.900	0.028	0.011	0.066	0.034	0.010	0.005	0.937
Gender										
Male		275.3^b^	73.58^a^	1.430^b^	0.837^a^	3.733^b^	1.980	0.362^b^	0.068^b^	77.83^a^
Female		316.7^a^	64.00^b^	2.155^a^	0.730^b^	6.189^a^	1.890	0.400^a^	0.104^a^	68.77^b^
SEM		3.186	0.900	0.028	0.011	0.066	0.034	0.010	0.005	0.937
Source of variation, *p-*value										
Quail strain		< 0.001	< 0.001	< 0.001	< 0.001	0.191	0.335	< 0.001	0.002	< 0.001
Gender		< 0.001	< 0.001	< 0.001	< 0.001	< 0.001	0.095	0.032	0.001	< 0.001
Quail strain × gender		0.114	0.079	0.015	0.582	0.003	0.018	0.282	0.253	0.120

Means in the same column within each classification bearing different letters are significantly different

*SEM* standard error of means

### Breast muscle characteristics

The investigation of the breast muscle characteristics and collagen content of breast muscle in two Japanese quail strains (white vs. gray) is illustrated in Table 4. The results of WJQS indicated higher weight values for the right pectoralis major muscle (PC) (23.39 vs. 20.37 g, *P* 0.004), left PC (23.31 vs. 20.07 g, *P* 0.004), right supracoracoideus muscle (SC) (7.29 vs. 5.83 g, *P* ˂ 0.001), and left SC (8.1 vs. 5.57 g, *P* ˂ 0.001) than the GJQS. Furthermore, sex differences were observed inside strain; females of WJQS showed a higher value of SC and PC muscles than males, while males of GJQS showed a higher value of SC and PC muscles than females.

**Table 4:** Differences in collagen type 1 concentration in breast tissue and absolute weight (AW), relative weight (RW) of pectoralis (PC), and supracoracoideus (SC) muscles in jumbo and gray Japanese quail strains

Indices		Right PCAW	Right PCRW	Right SCAW	Right SCRW	Left PCAW	Left PCRW	Left SCAW	Left SCRW	Collagen 1 (Pg/ml)
Quail strain	Gender									
Jumbo	Male	22.19^b^	8.179	6.914^b^	2.497	21.26^b^	7.691	8.075	2.916	238.3^c^
Jumbo	Female	24.60^a^	8.022	7.675^a^	2.553	25.36^a^	8.426	8.131	2.705	137.3^d^
Gray	Male	21.30^ab^	8.401	6.366^c^	2.511	20.38^b^	8.043	5.439	2.145	636.0^a^
Gray	Female	19.45^c^	7.771	5.303^d^	2.114	19.75^b^	7.871	5.714	2.291	422.3^b^
SEM		0.711	0.220	0.312	0.075	0.864	0.213	0.440	0.126	57.96
Quail strain										
Jumbo		23.39^a^	8.100	7.294^a^	2.525	23.31^a^	8.059	8.103^a^	2.810^a^	187.8
Gray		20.37^b^	8.086	5.834^b^	2.312	20.07^b^	7.957	5.576^b^	2.218^b^	529.2
SEM		0.506	0.379	0.114	0.097	0.553	0.366	0.280	0.157	
Gender										
Male		21.74	8.212	6.640	2.504	20.82^b^	7.867	6.757	2.530	437.2
Female		22.03	7.975	6.489	2.333	22.55^a^	8.149	6.922	2.498	279.8
SEM		0.438	0.328	0.099	0.084	0.479	0.317	0.243	0.136	14.56
Source of variation, *p-*value										
Quail strain		0.004	0.978	< 0.001	0.149	0.004	0.841	< 0.001	0.029	< 0.001
Gender		0.683	0.653	0.357	0.233	0.005	0.582	0.671	0.882	< 0.001
Quail strain × gender		0.019	0.462	0.001	0.129	0.018	0.385	0.777	0.424	0.026

Means in the same column within each classification bearing different letters are significantly different

*SEM* Standard error of means

#### Genetic estimations

Microsatellite loci, annealing temperatures, primer sequence, gene bank accession numbers, repeat array, and band size are shown in Table 1. Annealing temperatures ranged from 48 with UBC001 to 64 with GUJ0053; the band size ranged from 35 to 610 bp in WJQS and GJQS with fourteen microsatellite markers, as shown in Table 1.

The total number of alleles was 97 out of fourteen microsatellite markers ranged from 3 with GUJ0013 and GUJ0051 to 23 with UBC001 in WJQS and GJQS. The total number of alleles per strain was 62 ranged from one in GUJ0048 to 12 in UBC001 with a mean of 4.43 in WJQS while, the total number of alleles was 70 ranged from two in GUJ0028, GUJ0053, and GUJ0054 to 12 in UBC001 with a mean of 5.00 in Japanese quails strain as shown in Table 5. Regarding specific alleles, a total of 62 out of 97 alleles (63.92%) were detected overall loci (14 microsatellite loci) versus two strains. For WJQS, 27 with a mean of 1.93 specific alleles were observed, while 35 with a mean of 2.50 were obtained for GJQS. These specific alleles would be utilized as a strain fingerprint in WJQS and GJQS.

**Table 5:** Number of alleles observed for each locus within each quail strain, total no. of alleles, specific alleles, observed (HO) and expected (HE) heterozygosities, effective number of alleles (ENA), and similarity between jumbo and gray Japanese quail strains

Locus	No. of alleles per strain		No. of specific alleles		Total no. of alleles per locus	Observed heterozygosities (HO)	Expected heterozygosities (HE)	Effective no. of alleles (ENA)	% similarity between jumbo and gray
	Jumbo	Gray	Jumbo	Gray					
GUJ0013	2	3	--	1	3	0.49	0.6	2.4522	66.67
GUJ0021	3	4	--	1	4	0.05	0.66	2.8832	75.00
GUJ0028	3	2	3	2	5	0.11	0.77	4.1734	00.00
GUJ0048	1	4	--	3	4	0.33	0.3	1.4307	25.00
GUJ0051	3	3	--	--	3	0.24	0.49	1.9310	100.00
GUJ0052	5	7	2	4	9	0.27	0.82	5.2205	33.33
GUJ0053	3	2	1	--	3	0	0.26	1.3536	66.67
GUJ0054	5	2	4	1	6	0.03	0.75	3.7766	16.67
GUJ0057	5	7	4	6	11	0	0.89	8.0476	9.09
GUJ0087	4	5	--	1	5	0.02	0.78	4.3047	80.00
GUJ0099	7	5	2	--	7	0.1	0.83	5.4953	71.43
UBC001	12	12	11	11	23	0.85	0.96	17.9775	4.35
UBC002	6	8	--	2	8	0.02	0.83	5.4845	75.00
UBC005	3	6	--	3	6	0.05	0.67	2.9935	50.00
Total	62	70	27	35	97				
Mean	4.43	5.00	1.93	2.50	6.93			4.82	48.09

**Fig. 1:**
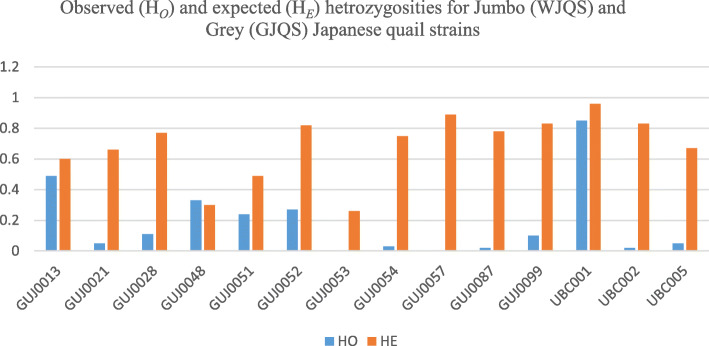
Observed (H_*O*_) and expected (H_*E*_) hetrozygosities for jumbo (WJQS) and gray (GJQS) Japanese quail strains

Specific alleles for the WJQS strain could not be detected using the markers GUJ0013, GUJ0021, GUJ0048, GUJ0051, GUJ0087, UBC002, and UBC005. Also, GUJ0051, GUJ0053, and GUJ0099 produced no specific alleles for Japanese strain, as shown in Table 5. In respect to ENA, it was used to corollary detect the expected heterozygosity (HE) where the effective number of alleles is the highest when heterozygosity is high. In our results, the lowest ENA was 1.35 for GUJ0053 when HE was 0.26 while the highest ENA was 17.98 for UBC001 when HE was 0.96 (Table 5 and Fig. 1). The degree of genetic variation of the microsatellite loci was reflected by heterozygosity in strains. Also, high heterozygosity indicated a high genetic diversity as well as a high degree of genetic variation. Out of 14 microsatellite sequences selected for detecting the differentiation and similarity between WJQS and GJQS, the percentage of similarity was 48.09% ranged from 4.35 with UBC001 to 100% with GUJ0051. The highest number of alleles per strain, the specific alleles, the total number of alleles, and the significant number of alleles were detected in UBC001, which had 12 numbers of alleles per strain and 11 specific alleles within the two strains and a total number of 23 alleles with ENA was 17.98, and the lower percentage of similarity was 4.35.

**Fig. 2:**
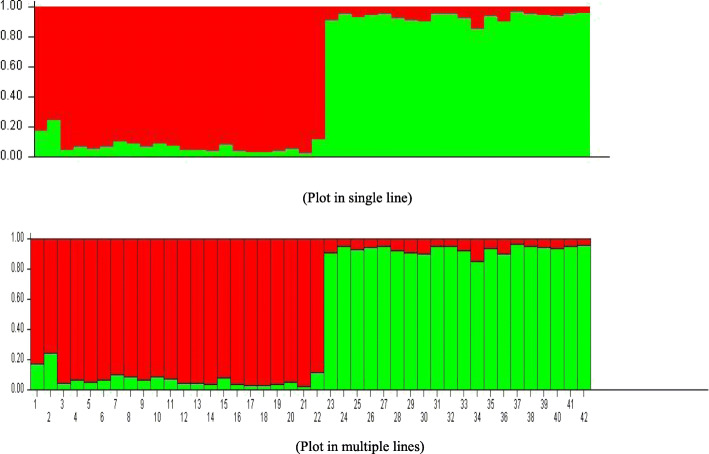
Bar plot from structure results using *K* = 2 clusters. Group 1 = 1–22 samples Jumbo (WJQS) and Group 2 = 23–42 samples gray (GJQS) Japanese quail strains; each vertical line represents the proportion of origin (q) of an individual in the first (green, GJQS) and second (red, WJQS) cluster

The estimated proportions of WJQS for each individual are represented by the green bar’s length, as shown in Fig. 2. The red bars in the group indicate that several hybrids and probably even pure Japanese individuals (whole red bar) are present in the GJQS.

Allelic frequencies were calculated based on all fourteen microsatellite loci. The highest allele frequency overall loci were 1.00 for allele 80 at GUJ0048 locus in WJQS, while the lowest one (0.023) was for allele 65 at GUJ0051 locus in WJQS. Also, the highest average of allele frequency estimated was 0.33 at loci GUJ0013 and GUJ0053. Meanwhile, the lowest one was 0.04 at locus UBC001.

Polymorphic information content (PIC) refers to the possibility that a progeny acquires some allelic markers from its father or mother, describing the variation degree of microsatellite loci. The value of PIC for WJQS ranged from 0.19 to 0.89 in GUJ0053 and UBC001, with a mean of 0.58 in WJQS. While it ranged from 0.32 to 0.89 in GUJ0053 and UBC001 with a mean of 0.62 in GJQS as shown in Table 6, these differences reflect high genetic variability between two quail strains.

**Table 6:** Specific alleles in base pairs and frequencies observed for jumbo and gray Japanese quail strains.

		Frequency					Frequency		
Locus	Alleles	common alleles		Specific alleles	Locus	Alleles	Common alleles		Specific alleles
GUJ0013		Jumbo	Gray		GUJ0021		Jumbo	Gray	
	80	0.619	0.375			155	0.000	0.722	Gray
	90	0.381	0.375			166	0.666	0.222	
	100	0.00	0.250	Gray		177	0.167	0.028	
Average		0.33	0.33			188	0.167	0.028	
PIC		0.47	0.66		Average		0.25	0.25	
GUJ0028	104	0.278	0.000	Jumbo	PIC		0.50	0.43	
	113	0.444	0.000	Jumbo	GUJ0048	52	0.000	0.325	Gray
	122	0.278	0.000	Jumbo		66	0.000	0.025	Gray
	158	0.000	0.700	Gray		80	1.000	0.625	
	167	0.000	0.300	Gray		94	0.000	0.025	Gray
Average		0.20	0.20		Average		0.25	0.25	
PIC		0.65	0.42		PIC		0.00	0.50	
GUJ0051	45	0.613	0.650		GUJ0052	55	0.333	0.000	Jumbo
	55	0.364	0.325			67	0.548	0.000	Jumbo
	65	0.023	0.025			79	0.071	0.375	
Average		0.33	0.33			91	0.024	0.325	
PIC		0.49	0.47			103	0.024	0.075	
GUJ0053	177	0.900	0.800			115	0.000	0.025	Gray
	196	0.050	0.200			127	0.000	0.075	Gray
	215	0.050	0.000	Jumbo		139	0.000	0.100	Gray
Average		0.33	0.33			151	0.000	0.025	Gray
PIC		0.19	0.32		Average		0.11	0.11	
GUJ0054	54	0.000	0.474	Gray	PIC		0.58	0.73	
	61	0.277	0.526		GUJ0057	130	0.000	0.050	Gray
	68	0.278	0.000	Jumbo		142	0.000	0.050	Gray
	75	0.139	0.000	Jumbo		154	0.000	0.350	Gray
	82	0.278	0.000	Jumbo		166	0.000	0.100	Gray
	103	0.028	0.000	Jumbo		178	0.000	0.200	Gray
Average		0.17	0.17			190	0.000	0.200	Gray
PIC		0.75	0.50			202	0.210	0.050	
GUJ0087	154	0.159	0.052			214	0.105	0.000	Jumbo
	165	0.273	0.316			226	0.316	0.000	Jumbo
	176	0.318	0.158			238	0.316	0.000	Jumbo
	187	0.250	0.263			250	0.053	0.000	Jumbo
	198	0.000	0.211	Gray	Average		0.09	0.09	
Average		0.20	0.20		PIC		0.74	0.78	
PIC		0.74	0.76		GUJ0099	35	0.182	0.000	Jumbo
UBC001	475	0.000	0.025	Gray		42	0.046	0.000	Jumbo
	480	0.050	0.000	Jumbo		49	0.273	0.050	
	485	0.000	0.100	Grey		56	0.227	0.275	
	490	0.150	0.000	Jumbo		63	0.046	0.425	
	495	0.000	0.100	Gray		70	0.182	0.125	
	500	0.050	0.000	Jumbo		77	0.046	0.125	
	505	0.000	0.100	Gray	Average		0.14	0.14	
	510	0.175	0.000	Jumbo	PIC		0.80	0.71	
	515	0.000	0.100	Gray	UBC002	190	0.000	0.0250	Gray
	520	0.025	0.000	Jumbo		211	0.1905	0.3500	
	525	0.000	0.100	Grey		218	0.2857	0.1250	
	530	0.100	0.000	Jumbo		225	0.1905	0.1000	
	535	0.000	0.025	Gray		232	0.1905	0.1000	
	560	0.025	0.025			239	0.0476	0.0500	
	565	0.050	0.000	Jumbo		246	0.0952	0.2000	
	570	0.000	0.075	Gray		253	0.000	0.0500	Gray
	575	0.100	0.000	Jumbo	Average		0.13	0.13	
	580	0.000	0.175	Gray	PIC		0.80	0.80	
	585	0.150	0.000	Jumbo	UBC005	100	0.000	0.0526	Gray
	590	0.000	0.125	Gray		109	0.0952	0.2105	
	595	0.100	0.000	Jumbo		118	0.6190	0.1579	
	605	0.000	0.050	Gray		127	0.2857	0.5000	
	610	0.025	0.000	Jumbo		136	0.000	0.0526	Gray
Average		0.04	0.04			181	0.000	0.0263	Gray
PIC		0.89	0.89		Average		0.17	0.17	
					PIC		0.53	0.67	
Mean PIC	Jumbo	0.58							
	Gray	0.62							

*PIC* polymorphic information content

In this study, three markers GUJ0013 (0.47), GUJ0051 (0.49), and GUJ0053 (0.19) were reasonably informative (0.50 > PIC>0.25). Marker of GUJ0048 (0.00) was a slightly informative marker, and most of the loci were highly informative with WJQS. Four markers GUJ0021 (0.43), GUJ0028 (0.42), GUJ0051 (0.47), and GUJ0053 (0.32) were reasonably informative (0.50 > PIC>0.25), while the majority of the loci were highly informative (PIC ≥ 0.50) with GJQS. The analysis of molecular variance estimated by the Arlequin 3.5 software package as standard genetic strain input data is presented in Table 7. Variance components proved that most genetic diversity obtained in the current study is represented within individuals (24.11%) rather than others. Fixation indices give an idea about the strain’s structure in terms of straining coefficient and strain differentiation. Strain fixation indices traced a 0.759 variation, referring to differences among individuals versus total variance (FIT). While among strains, differences versus total variance were the lowest fixation indices (FST = 0.178), indicating a low level of strain differentiation. These observations might be explained as approximate equality of the average total number of alleles detected for each strain overall loci. It was 4.43 for WJQS and 5.00 for GJQS, as shown in Table 7.

**Table 7:** ANOVA analysis of jumbo and gray Japanese quail strains based on microsatellite DNA variation

Source of variation	d.f	S.S	Percentage variation	Fixation indices
Among strains	1	44.04	17.812	F_*IS*_ = 0.70669
Among individuals within strains	40	282.26	58.081	F_*ST*_ = 0.17812
Within individuals	42	52.00	24.11	F_*IT*_ = 0.75893
Total	83	378.31		

*FIS* Fixation indices (among strains), *FST* Fixation indices (among individuals within strains), *FIT* Fixation indices (within individuals), *d.f* Degrees of freedom, *S.S* Sum of squares

## Discussion

### Physiological estimation

The significant differences in the two-color variants studied (WJQS and GJQS) in the marketing body weight and different body measurements such as body length, chest girth, chest length, thigh length, thigh girth, drumstick length, and drumstick girth reflect the differences between WJQS and GJQS in body sizes and shape, indicating positive relationships between body weights and body measurements (Tables 5 and 6). Moreover, the obtained results confirm the physiological variations between WJQS and GJQS, which may be due to the existence of genetic variation between them. The obtained results agree with several workers that reported a positive correlation between live body weight and morphometric body measurements in Isa Brown and Ilorin ecotype chickens [20], in two commercial broiler strains [21], in Japanese Quails [22], in the French broiler guinea fowl [23], and two commercial meat-type chickens [24]. Moreover, it is well-known that body weight is considered the most important physiological indices for evaluating different livestock species for numerous reasons, including its relation with body growth and other physiological traits such as body morphometric measurements, carcass characteristics, and breast meat yield. In an overall comparison of two quail strains, the WJQS attained greater physiological parameters in terms of body weight, carcass yield, most of the body organs, and breast meat yield than GJQS. This might be attributed to the superior genetic potential of WJQS than GJQS, which lead to higher marketing bodyweight and produced more massive carcass and more meat. These observations are consistent with Ojedapo et al. [21] and Ahmad et al. [22] who reported a strong genetic correlation between body weight and carcass traits. Similarly, other studies [25–27] reported higher carcass yield in selected heavy lines of Japanese quail superior to that of a non-selected.

Furthermore, it is generally accepted that both PC and SC muscles are positively correlated with marketing body weight, muscle mass, and meat quality. In this mention, Młynek et al. [28] showed that dressing percentage significantly affected carcass and pectoralis significant muscle weight and soluble collagen. Therefore, the selection for increased live body weight in earlier studies performed by Ryu et al. [29] and Rehfeldt et al. [30] was a suitable way to enhance Japanese quail’s growth performance. Baylan et al. [31] reported a similar finding; Anjum et al. [32] observed a higher breast meat yield in birds selected for body weight. In another explanation, Choi et al. [33] reported a positive correlation between DNA contents and muscle weights between quail lines. The present study’s results sustain this finding regarding the collagen content of breast muscle in WJQS and GJQS. Significant differences were found: breast muscles of GJQS exhibited a higher concentration of type I collagen, almost three-fold than the WJQS (529.2 vs. 187.8 Pg/ml, *P* < 0.001), respectively (Table 7). Furthermore, sex differences were observed inside each strain; the male showed a higher value of collagen type I concentration than the female (437.2 vs. 279.8 Pg/ml, *P* < 0.001), respectively. It is well-known that intramuscular collagen is an essential parameter to the meat industry; an increased amount of this component may affect toughness and meat quality. In other words, the most abundant fibrous form of collagen in muscle is type I, which considers the main structural protein of connective tissues present in meat, providing meat toughness and rigidity and involved in the structural integrity and several physiological functions [34, 35]. Moreover, the significant factor affecting meat tenderness is the maturity of connective tissues, which is a function of chemical cross bonding of the collagen in the muscle, which increases with age; hence, the tough meat is found in older birds [36]. Therefore, the differences in collagen content in WJQS and GJQS, which feed on the same diet and under the same age, may confirm the existence of genetic variations between them.

### Genetic estimations

Examining the results of the 14 microsatellite markers in this work showed some genetic differences in two quail strains WJQS and GJQS. As a result, the observed genetic differences confirmed the presence of physiological variations between WJQS and GJQS, such as body weight, carcass characteristics, body measurements, breast muscle weights, and collagen type I concentration of breast muscle. Our results are consistent with similar studies conducted by Charati et al. [16] and Moradian et al. [17], which showed the relation of these locations with cold carcass weight, breast meat weight, and body dimensions and carcass parameters. The obtained results on annealing temperatures and the size of bands in two strains (WJQS and GJQS) with 14 microsatellite markers agree with Roushdy and El-Sayed’s [9] results from 60 to 470 bp with UBC001, UBC002, UBC005, and GUJ0028. Also, it agrees with Kayang et al. [15], wherein the values ranged from 96 to 284 bp. Besides, the mentioned obtained values could be informative for such studies, according to Kawahara-Miki et al. [37], who suggested that the allele sizes of the DNA fragments for the 101 markers ranged from 7 to 36 repeats and 91 to 311 bp, respectively, in the Japanese quail, while Bai et al. [38] observed that the annealing temperatures ranged from 46 to 58 with 12 microsatellite markers. Moreover, the total number of alleles per strain agreed with Kayang et al. [15], who reported that the average of 1.9 alleles per locus ranged from one to four alleles. Also, Choi et al. [39] reported that the mean number of alleles in each breed ranged from 3.59 to 6.63. Further studies carried on different quail genotypes by Bai et al. [40], Kawahara-Miki et al. [37], Bai et al. [38], Shimma and Tadano [41], and Habimana et al. [10] reported the allele size of 48, 70, 197, 308, and 305, respectively. Furthermore, the specific alleles for WJQS were 27 and 35 with GJQS. These values were lower than those observed by Roushdy and El-Sayed [9], who detected 68 out of 136 specific alleles (50%) overall loci (12 microsatellite loci) versus two species. Also, Habimana et al. [10] showed 20% of private alleles. A high value of heterozygosity (51.91%) between two quail strains with 14 microsatellite loci and the effective number of alleles that ranged between 1.6504 (MCW0078) and 8.901 (LEI0234) indicated the relatively rich genetic variation of two strains and a significant genotype of WJQS than GJQS. However, ENA’s obtained value was more significant than the estimated value reported by Habimana et al. [10]. On the other side, the allele frequency over all loci ranged from 0.023 to 1.00 with WJQS. These results agree with El-sayed [42], who reported that the specific allele frequency value ranged from 0.05 to 0.50 based on 15 microsatellite loci used for Fayoumi and Dandarawi breeds. When PIC values were examined, it was seen that a substantial portion of working locus markers provided information at a high level. When Table 3 was analyzed in terms of PIC means, the value was highly informative (PIC ≥ 0. 50); it was observed that there was a difference among quail strains. According to the classification of Botstein et al. [43], the highly informative markers have PIC values > 0.50, the reasonably informative markers have a PIC value between 0.25 and 0.50, and the slightly informative markers have PIC value less than 0.25. In this study, three markers GUJ0013 (0.47), GUJ0051 (0.49), and GUJ0053 (0.19) were reasonably informative (0.50 > PIC > 0.25). Marker of GUJ0048 (0.00) was a slightly informative marker. The majority of the loci were highly informative with WJQS. Four markers GUJ0021 (0.43), GUJ0028 (0.42), GUJ0051 (0.47), and GUJ0053 (0.32) were reasonably informative (0.50 > PIC > 0.25), while the majority of the loci were highly informative (PIC ≥ 0. 50) with GJQS. This suggests that a high degree of polymorphism has potentially been maintained in two strains WJQS and GJQS. Also, Bai et al. [38] reported that the average PIC of 12 microsatellite markers at Chinese yellow quail, Chinese black quail, and Korean quail which are 0.6853, 0.6401, and 0.6565 respectively were highly informative (PIC ≥ 0.50). Habimana et al. [10] showed that the PIC ranged from reasonably to highly informative since the PIC for the loci MCW0103 and LEI0234 were 0.3488 and 0.8775, respectively. Fixation indices give an idea about the strain’s structure in terms of straining coefficient and strain differentiation. Also, the investigation had been done by Vargas et al. [44] who reported that FIS ranged from a minimum of − 0.034 (MCW014) to a maximum of 0.727 (MCW014) with an average of 0.146 (0.1254–0.1638). Finally, Habimana et al. [10] showed that the contribution of 28 microsatellites for population segregation (determined by FST statistics) varied from 0.000 (MCW0037) to 0.158 (ADL0268).

## Conclusion

This study showed highly physiological differences between WJQS and GJQS in live body weight, carcass characteristics, body measurements, breast muscle weights, and collagen type I concentration of breast muscle. These physiological variations were ascertained with selected 14 microsatellite markers, which indicated the relatively rich genetic variation of the two strains and a significant genotype of WJQS than GJQS. These results succeeded in introducing a scientific basis for the evaluation and utilization of genetic resources of WJQS and GJQS in the next breeding programs for genetic improvement of the breed in an attempt to stop the continuous inbreeding system in quail farming and, consequently, improve the production performance of Japanese quail.

## Data Availability

Not applicable

## Abbreviations

PIC: Polymorphism information content
PCR: Polymerase chain reaction
HO: Observed heterozygosities
HE: Expected heterozygosities
ENA: Effective number of alleles
FIS: Fixation indices (among strains)
FST: Fixation indices (among individuals within strains)
FIT: Fixation indices (within individuals)
IC: Inbreeding coefficient
D.F: Degrees of freedom
S.S: Sum of squares
GGA, CJA, and QL: Linkage group chicken and Japanese quail chromosome
